# A risk-based decision framework for policy analysis of societal pandemic effects

**DOI:** 10.3389/fpubh.2023.1064554

**Published:** 2023-02-17

**Authors:** Mats Danielson, Love Ekenberg, Nadejda Komendantova, Adriana Mihai

**Affiliations:** ^1^Department of Computer and Systems Sciences, Stockholm University, Stockholm, Sweden; ^2^Advancing Systems Analysis (ASA), International Institute for Applied Systems Analysis (IIASA), Laxenburg, Austria

**Keywords:** policy formation, policy analysis, decision analysis, MCDM, imprecise probabilities

## Abstract

**Introduction:**

In this article, we summarize our findings from an EU-supported project for policy analyses applied to pandemics such as Covid-19 (with the potential to be applied as well to other, similar hazards) while considering various mitigation levels and consequence sets under several criteria.

**Methods:**

It is based on our former development for handling imprecise information in risk trees and multi-criteria hierarchies using intervals and qualitative estimates. We shortly present the theoretical background and demonstrate how it can be used for systematic policy analyses. In our model, we use decision trees and multi-criteria hierarchies extended by belief distributions for weights, probabilities and values as well as combination rules to aggregate the background information in an extended expected value model, taking into criteria weights as well as probabilities and outcome values. We used the computer-supported tool DecideIT for the aggregate decision analysis under uncertainty.

**Results:**

The framework has been applied in three countries: Botswana, Romania and Jordan, and extended for scenario-building during the third wave of the pandemic in Sweden, proving its feasibility in real-time policy-making for pandemic mitigation measures.

**Discussion:**

This work resulted in a more fine-grained model for policy decision that is much more aligned to the societal needs in the future, either if the Covid-19 pandemic prevails or for the next pandemic or other society-wide hazardous emergencies.

## 1. Introduction

The societal reactions to the Covid-19 pandemic, which did affect all countries worldwide, resulted in many incongruences in risk-reduction processes and policies. Public health emergencies made the policy decisions less a result of deliberations taking various risks and stakeholders into consideration, and the responses were more a product of uncertain projections implemented in a top-down fashion. These left many risks, preferences, and consequences unaddressed and unmodelled (1), an oversight that has often been justified by blaming the inherent uncertainty of epidemiologic data. Even though more recent attempts to devise future pandemic scenarios make more efforts in taking uncertainty into consideration when conceptualising policy approaches and assessing risk, there is still a lack of structure and methods in how to effectively integrate imprecise information in pandemic response strategies. In this article, we address this issue by suggesting an entire framework for policy analyses containing several different criteria and made under severe uncertainty, that is used to analyse pandemic effects. This is addressed while considering epidemiologic estimations and socio-economic factors in a multi-stakeholder and multi-criteria context, based on a process for eliciting attitudes and perceptions as well as preferences amongst different stakeholder groups. The model was used to structure the analysis of pandemic effects, as well as to evaluate the effects of various response strategies to hazardous events, such as the Covid-19 pandemic, and to mobilise better mitigation measures for future scenarios related to pandemics and other hazardous events.

Prevailing decision-making methods under uncertainty are quite limited when reliable data is scarce or entirely missing, which is invariably the case when modelling an ongoing pandemic. Uncertainty about policy impacts leads to much higher socioeconomic costs, creating possibly undesired and expensive side effects concerning the impacts of the choices of relevant policies across a variety of interconnected sectors. In our model, we do not only investigate epidemiologic and healthcare factors separately, but our approach and main contribution to existing decision mechanisms on pandemic responses is to cover the entire policy problem and to include a variety of factors that are necessary for policy responses to such events.

The article thus presents the conceptual framework and formalisation of policy-making in conditions of uncertainty during the pandemic, summarising our findings from an EU-supported project for policy analyses (2), and then showcases how it has been extended since, by analysing Sweden's decision-making on future mitigation strategies depending on vaccination efficiency during the third wave of the pandemic. Our conceptual framework for decision analysis under uncertainty was previously applied in three countries: Botswana (3), Romania (1) and Jordan (4, 5), where data collection and stakeholder consultation processes provided the input for the modelling and evaluation. While that framework was a best effort during the peak of the pandemic, the analysis model has now been considerably extended by taking different scenario outcomes and their probabilities into account. Thus, the resulting probabilistic model is more fine-grained and enables a more precise basis for societal policymaking during pandemics and other similar society-wide crises.

The next section discusses policy formation at large and a literature review of the problems raised by uncertainty in policy making in the context of the Covid-19 pandemic. Then, responding to these issues, we introduce a method for integrating imprecise information in combined risk trees and multi-criteria hierarchies and how it can be used in systematic policy analysis. In section four, we present the decision theoretical framework and demonstrate how it can be used in systematic analyses, which can serve pandemic mitigation policy making. Whereafter, we further discuss the utility and implications of our result, namely a model for societal policies to counter hazardous events from a policy point of view, while considering criteria, such as epidemiologic estimates and socioeconomic factors. This is best managed, we conclude, in a multi-stakeholder and multi-criteria context, based on a co-creative work process for eliciting attitudes, perceptions, as well as preferences among stakeholder groups.

## 2. A case for systematic analysis of policy formation

The prevailing approach in policy-making for mitigating the pandemic effects has been evidence-based policy making, where governments consulted with experts and relied on epidemiologic modelling in order to devise scenarios and actionable measures. In this approach, several problems became apparent when dealing with an emergency situation: time pressure affected planning, the availability of resources determined the feasibility of various actions and the uncertainty of evidence itself paved the way to uncertain projections that could not provide a clear-cut direction for policymakers. Schippers Michaéla and Rus Diana (6) discussed the errors and biases that affected information processing in, among others, defining the decision problem in a much too narrow domain that failed to address spillover effects on societal and economic areas. Policymakers framed the problem as a short-term, quick reaction to subsequent emergencies (7), their purposes changing from protecting healthcare capacity to protecting vulnerable groups to, ultimately, protecting the economy. In evidence-based policy making, the limitation of what constitutes “evidence” considering the bias in knowledge assessment (8) was doubled by the unilateral understanding of the policy problem initially framed as a health emergency alone.

Since then, the plethora of unintended and unmodelled consequences upon other policy areas and socio-economic domains across the world made it necessary to look for other policy approaches that can supplement governments' efforts to obtain optimal results that produce little to no costly effects upon areas outside of the health crisis while managing the pandemic. Su (9), for example, provides a review of available policy-making strategies that can work in pandemic mitigation responses, reaching a conceptual model (PADS) that includes people-centred policymaking, decision-making mechanisms that are data-driven and supported by artificial intelligence, and a close supervision of the decision process. While this model acknowledges that collaboration with citizens and stakeholders is key to the policy-making cycle, it does not include any strategy on how to integrate uncertainty and multi-criteria impact assessments upon other interconnected domains, neither conceptually, nor formally. Better support for decision-making mechanisms has been sought elsewhere as well (10), but aside from the need to recognise uncertainty of input parameters and variations in process, no suggestion on how to formalise this within the policy decision models is made. Using modern decision theory to formalise policymaking under conditions of uncertainty has been suggested in Berger et al. (11) so as to clarify the decision problem, to incorporate trade-offs, to avoid reasoning mistakes and biases and to hold accountability; it remains unclear however, as the authors point out, how to establish decision criteria and how to aggregate stakeholder preferences in the available models.

More recently, a call to use a systems approach in order to point decision-makers towards seeing how one decision taken in one dimension will have impacts upon other policy dimensions has been launched by the Covid-19 Outcome Scenarios Project (12), which signals the uneven responses and cascading impacts of previous policy approaches adopted during the pandemic. The authors suggest three future scenarios for the Covid-19 pandemic and analyse them across a systems map that takes into consideration several outcome domains, policy dimensions or “clocks” and vectors of uncertainty that interact and affect mid- and long-term impacts of the pandemic. The vectors of uncertainty set out by the experts included in the study are, among others, global access to effective vaccines, reduction of social inequalities, income equality as well as the level of multilateral cooperation and the regional and global geopolitics. Aside from conceptualising a systems approach that builds upon inter-linked policy dimensions and uncertainties, the authors also point out that in order to increase resilience in such policy complexity, policymakers need to start integrating risk assessment into policy development. In our study, we adopt a similar viewpoint while we furthermore provide a decision-making mechanism and method through an integrated framework that includes, alongside multiple domains and criteria and evaluation under risk, stakeholder preferences as well.

The systematic analysis of policy formation usually focuses on system-level transformations and key societal transitions for societal resilience and sustainable systems through an enhanced understanding of, and the ability to manage existing challenges. Transformative governance aims to provide incentives, guidelines, methods, and tools for balancing efficiency, redundancy, uncertainty, and indeterminacy, as well as supporting policy-making on complex issues where several alternatives and trade-offs are possible. Our research will take into account the complexity of interconnected socio-economic and environmental systems, especially for policies trade-offs issues as well as existing uncertainties and heterogeneous social values regarding various relevant criteria. The focus is also on existing and emerging governance challenges and their complex structural dynamic evolutions, including multiple causes and effects with feedback mechanisms, such as health-related issues including the Covid-19 pandemic.

The systemic analysis of policy formation should also focus on existing and emerging policy processes and governance mechanisms of adaptation to challenging contexts, as well as on engagement and acceptability of decision-making processes and their outcomes by various stakeholder groups. This also includes the development of strategies and mechanisms for their implementation, based on identified trade-offs between various criteria, various patterns of cooperation, integrated evaluations, and compromise solutions.

One of the important aspects of systemic analysis of policy formation is to provide research on institutional and governance structures for the development and implementation of contested policy solutions and on bridging the gap between science and policy. This includes participatory modelling, involving stakeholders and decision-makers, leading to decision support tools development while being responsive to their requirements. Therefore, co-creation, co-information, and co-design are also important aspects.

The systemic analysis also helps to identify the existing patterns of science-policy interaction, namely, how scientific assessments are provided as well as how decision support tools can contribute to the processes and how they respond to the actual needs of policy-makers; what is the path of transfer of these tools and assessment from science to policy; how various degrees of centralisation and decentralisation of decision-making processes influence the process of knowledge generation and transfer; how various stakeholders are involved in knowledge development and implementation of strategies, actions and policy-interventions based on its results; how policy interventions are influencing existing socio-economic and environmental challenges; and further on how scientific input can effectively be used. All these questions are relevant to the governance of Covid-19 or similar pandemics.

Another important aspect of systematic analysis of policy formation is its contribution to the development of feasible solutions that recognise the multi-objective nature of decision-making while developing integrated methods for handling various criteria and strategies as well as their effects in uncertain environments. This enables policy-makers to form robust decision bases and to include structural uncertainties. This also contributes to modelling realism by developing methods for decentralised decision-making while recognising the self-interests of agents and devising heterogeneity-specific and conflicting policies. Behavioural biases can be also incorporated while accounting for bounded rationality and imperfect foresight during decision-making processes at complex policy formation.

Therefore, this research contributes to policy design and governance of Covid-19 pandemic risks while collecting data from stakeholders' processes with the aim of supporting decision-making and incorporating systematic thinking into strategic policy planning as well as exploring several aspects of multi-criteria decision-making.

### 2.1. Mitigation measures and their effects

In the Covid-19 case, we worked with sets of mitigation strategies with some variations regarding transport and service restrictions and other conditions in place [see, for instance, the cases of Botswana (3); Romania (1); and Jordan (4, 5) for considerably more detailed accounts] that could be aggregated into different progressive levels of countermeasures, such as:

1) An unmitigated epidemic, except for pharmaceutical measures and case isolation.
2) Firmer local actions, such as closing schools/workplaces.
3) Personal protective and mild social distancing measures.
4) Imposed social distancing measures and restrictions on mobility in public places.
5) Partial lockdown—school closures, restaurants and large shopping centres closed, etc.

## 3. Multi-criteria decision making

**Figure 1:**
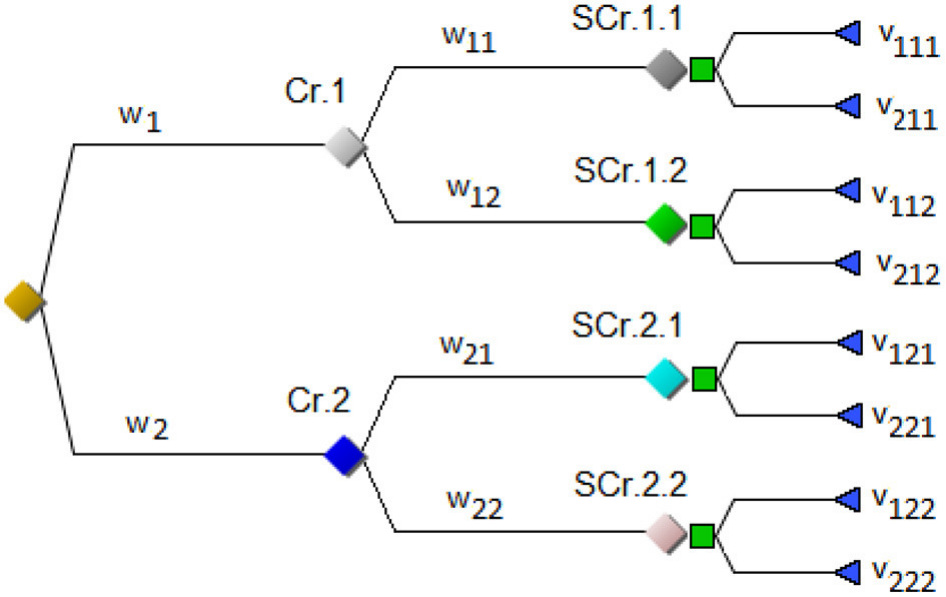
A multi-criteria hierarchy.

Multi-criteria decisions contain several criteria, sometimes in a hierarchy. In Figure 1, the alternatives are valued and the decision-maker assigns values to the alternatives. There is thus a set of criteria under which the various alternatives are considered. The possible courses of action to be taken are valued under each criterion and the relative importance of the criteria themselves is then represented by a set of weights that can be defined in several ways.

Weights are assigned to each sub-branch in the tree and the alternatives are valued under the respective sub-criteria. A weighted value is often used for evaluations. In Figure 1, w_1_ and w_2_ are the weights of the two main criteria Cr.1 and Cr.2 respectively, and they have to sum to 100%. For each of the two main criteria, there are two sub-criteria. For the first main criterion, the weights of the two sub-criteria SCr.1.1 and SCr.1.2 are w_11_ and w_12_, and for the second SCr.2.1 and SCr.2.2 have weights w_21_ and w_22_. Further, the figure contains a decision between two policy options, option 1 and option 2. Each option will have a consequence under each sub-criterion, leading to four consequences in total for each option. For option 1, the values of these consequences are v_111_, v_112_, v_121_, and v_122_ respectively. Thus, the first digit is the main criterion, the second is the sub-criterion and the third is the policy option. More formally, the value of alternative *A*_*i*_ under criterion *jk* is *v*_*ijk*_, while the weight of criterion *jk* is *w*_*jk*_. Thereafter, the total value of alternative *A*_*i*_ can be calculated using:


(1) $$\begin{array}{l} E\left(A_{i}\right)=\overset{2}{\underset{j=1}{\sum }}w_{j}\overset{2}{\underset{k=1}{\sum }}w_{jk}v_{ijk} \end{array}$$

where the alternative with the maximum expected value is then the preferred choice. This is the standard additive evaluation model of multi-criteria decision-making (MCDA) used by most decision analytic tools of today. What makes the current approach unique are three intertwined modelling features. The first is allowing subject experts as well as policymakers to state their assessment of criteria weights and outcome values as intervals instead of fixed numbers, i.e., not requiring unrealistic precisions in assessments but rather taking the levels of uncertainty into account. The second feature is the possibility to use rankings of criteria weights and outcome values, in case also numerical intervals are either too hard to estimate or politically sensitive. The model is still able to process the information and deliver an evaluation of the policy options. Finally, the model allows for mixing these modes of input as desired, making the best possible use of the available information at each point in time. See Section 4 for a continued discussion on modelling options.

In our case studies (Botswana, Jordan, and Romania), we worked with different criteria setups. For instance, a set of main criteria might have the following content:

a) Epidemiological and healthcare system effects;
b) Economic impact;
c) Social and behavioural aspects;
d) Political and governance.

For each main criterion (a)–(d), there can be sub-criteria such as those listed under (a)–(d) below. In real-life pandemic policy situations, it is desirable to have a finer granulation of the criteria, i.e., they are seen as composed of several components, called sub-criteria, which together make up the whole main criterion. As an example from one of our national policy models, the following constitutes the set of criteria, each divided into between 2 and 6 sub-criteria (1).

a) The criterion Epidemiological and healthcare system effects has two sub-criteria: direct fatalities and indirect fatalities;
b) The criterion Economic impact has 6 sub-criteria: short-term costs, unemployment, GDP growth, country development, taxes, and specific industries affected (including growing industries);
c) The criterion Social and behavioural aspects has five sub-criteria: human rights, protection of vulnerable groups, rates of criminality, mental health, and education and training;
d) The criterion Political and governance has four sub-criteria: risk of short-term governmental abuse, citizen approval of measures, trust in the government, and resilience (improving preparedness for catastrophic events).

The way this work is that for each main criterion, its sub-criteria are considered. This means that they are assigned weights according to how important they are relative to each other, with the sum of the weights adding up to 100%. In the next step, each main criterion as a whole is considered in comparison with the other main criteria, in this example four main criteria in total. They are assigned an independent set of weights such that also those sum to 100%. For example, assume that the sub-criteria for social and behavioural aspects have received the following weights. Human rights: 25%, protection of vulnerable groups: 15%, rates of criminality: 10%, mental health: 10%, and education and training: 40%. The same is then done with the other criteria. In the next step, the main criteria are weighted against each other. Assume that the weights at this upper level have been determined as (a) Epidemiological and healthcare system effects: 40%, (b) Economic impact 25%, (c) Social and behavioural aspects 20%, and (d) Political and governance 15%. Then while, for example, the sub-criterion human rights has a 25% weight locally (within its main criterion), it has a global weight (i.e., total influence in the policy analysis) of 40% – 25% = 10% since its parent is weighted at 40%.

## 4. An integrated framework

A basic premise is that policies are far from always determined by any scientific or economic rationality. There is a wide range of social, political, and other factors that interact to influence them and change them to social systems that, at best, assist in making deliberated and (hopefully) better public decisions. The social impact of having more deliberated analyses could also be to provide transparency and a more harmonised interaction between administrations and the public at large *via* the proposed modelling and analysis method which can address the needs of affected groups while providing better conformity between problems and solutions.

In many actual such decision situations, in which a structured model is to be employed, there are issues with estimating precise weights, probabilities and values for usage in the model and there have over the years been suggested a multitude of approaches to reduce this problem of artificial precision required, such as capacity theory, sets of probability measures, upper and lower probabilities, interval approaches, fuzzy measures and so on [cf. (13–15)]. These approaches, however, require a mathematical background on the part of the decision-makers. There are also complicated computational issues involved that we have studied over a long period of time [see, for example (16–19)]. For the cases in this study, we used the software DecideIT^1^ for integrated multi-attribute modelling and evaluation under risk (i.e., including probabilistic—Bayesian—modelling components), subject to incomplete or imperfect information in this framework. To avoid some aggregation problems when handling set membership functions and similar, we have over the years of research suggested a multitude of methods for analysing and evaluating decision problems with multiple stakeholders and multiple criteria. See, for instance, Komendantova et al. (20), Komendantova et al. (21), Komendantova et al. (22), Danielson and Ekenberg (23), Danielson et al. (19), Danielson et al. (5), and Fasth et al. (24) where we have suggested a set of evaluation methods for handling the information imprecision that is inevitably prevailing in policy decisions of the type addressed in this paper.

We argued in Section 3 for a model allowing severe uncertainty regarding the input information due to the circumstances when handling pandemics. Our earlier pandemic models, described in a previous Frontiers in Public Health paper (25), allowed for the imprecision described in Section 3. But there is another important category of uncertainty that has not hitherto been modelled in our pandemic policymaking processes. There is often more information available in these decision situations than only information on preferences among strategies for combating a pandemic. A natural extension of the model is to investigate the mitigation of the consequences in more detail. For instance, the fatality distributions for the scenarios contain more information regarding the probabilities for different outcomes. Such an extension can straightforwardly be integrated into the overall criteria structure of our model.

**Figure 2:**
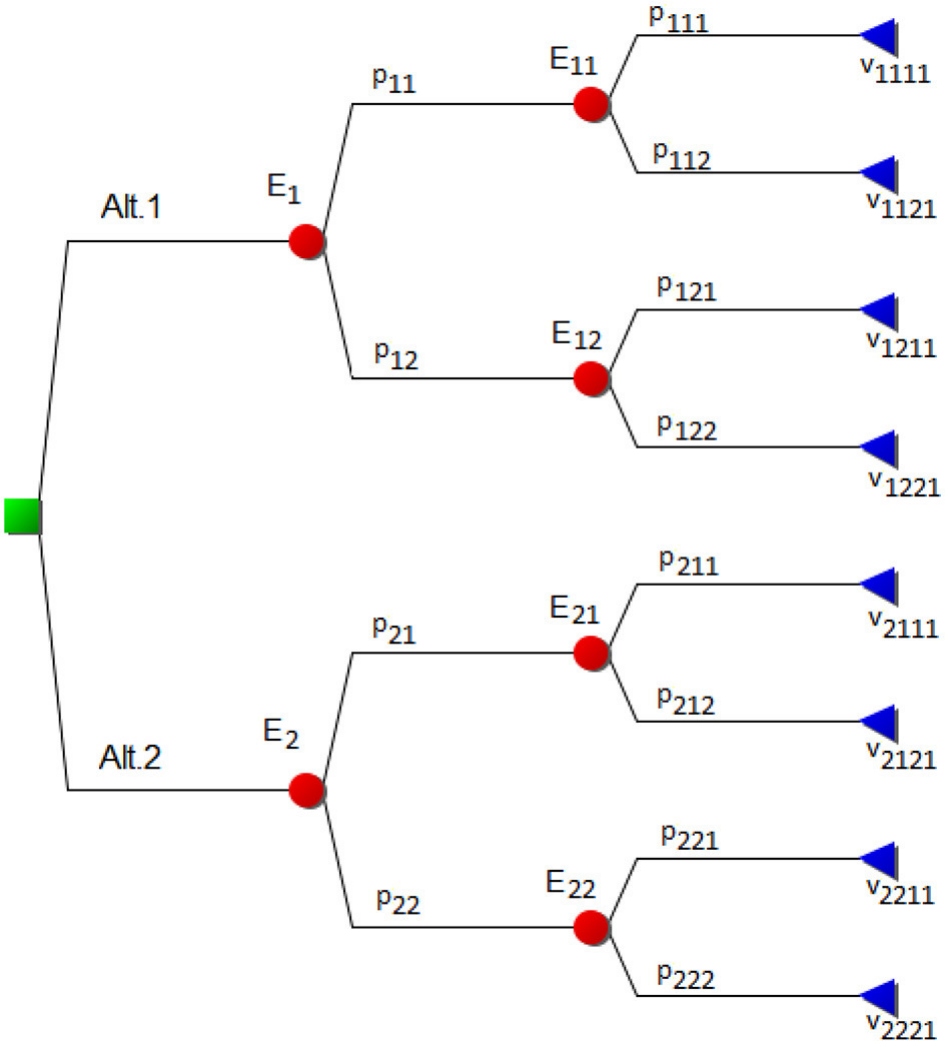
A decision tree with two alternatives.

Probabilistic decision situations are often represented by decision trees such as in Figure 2.

In the figure, there is a decision between two policy alternatives, Alt.1 and Alt.2. For each alternative, when selected one of two events will occur. For the first alternative, the probabilities for the outcomes of the event E_1_ are p_11_ and p_12_, while for the second they are p_21_ and p_22_ respectively. Next, after each of those events, a new event will occur as a consequence of the first event. Thus, we have two sets of secondary events and they might but need not be the same. Considering the follow-up events E_11_ and E_12_, they have the probabilities p_111_ and p_112_ for the first sub-event and p_121_ and p_122_ for the second, and analogously for the second main event E_2_. Finally, each chain of two events results in a consequence or state of the world, an outcome that is assigned a value. These values are, for the event with probability p_111_, labelled v_1111_, and for the event with probability p_112_, labelled v_1121_, and so on. The fourth digit signifies that this event decision tree belongs to criterion 1 in a multi-criteria decision analysis.

More generally, a decision tree consists of a root node, and two sets of nodes, representing uncertainty and outcomes, respectively. The probability nodes are assigned probability distributions representing uncertainties in the decision. When an alternative (or, more precisely, mitigation action in this case) *A*_*i*_ is chosen, there is a probability *p*_*ij*_ that an event will occur that leads to a subsequent event or a consequence. The consequences are assigned values *v*_*ijk*_. Here, the maximisation of the expected value is often used as an evaluation rule of the policy decision. For instance, the expected value of alternative *A*_*i*_ in the simplified event tree in Figure 2 is:


(2) $$\begin{array}{l} E\left(A_{i}\right)=\overset{2}{\underset{j=1}{\sum }}p_{ij}\overset{2}{\underset{k=1}{\sum }}p_{ijk}v_{ijk1} \end{array}$$

which can straightforwardly be generalised to trees of arbitrary depths and with any number of modelled consequences following an action.

Since the initial modelling during the Covid-19 pandemic was naturally hurried, it mostly contained a set of courses of action (levels of lockdown, rules and regulations for public spaces and public transport, etc.) and, at best, involved more than one criterion. If the models contained only one criterion, that was invariably the number of fatalities. But as we argue in Ekenberg et al. (1), there is much more to a policy decision situation, and an extended analysis is called for covering several criteria and, in many cases, sub-criteria. As more information becomes available as time goes by, the requirement increases to be able to incorporate this into the model and its evaluations. Most public health authorities started to create more fine-grained models with different scenarios for each policy strategy, and with accompanying uncertainties in the form of probabilities.

Co-evaluating the entire combined criteria weight/probabilistic decision problem is done by calculating the value of the alternatives in the criteria hierarchy. The expected value of the combined tree is then, for the simple case of combining Figures 1, 2, calculated by


(3) $$\begin{array}{l} E\left(A_{i}\right)=\overset{2}{\underset{j=1}{\sum }}\left(w_{j}\cdot \overset{2}{\underset{k=1}{\sum }}\left(w_{jk}\cdot \overset{2}{\underset{m=1}{\sum }}\left(p_{im}\cdot \overset{2}{\underset{n=2}{\sum }}p_{imn}v_{imn1}\right)\right)\right) \end{array}$$

or, in the general case, by


$$\begin{array}{l} E\left(A_{i}\right)=\overset{n_{j_{0}}}{\underset{i_{1}=1}{\sum }}w_{ii_{1}}\overset{n_{j_{1}}}{\underset{i_{2}=1}{\sum }}w_{ii_{1}i_{2\;}}\ldots \;\overset{n_{j_{m-2}}}{\underset{i_{m-1}=1}{\sum }}p_{ii_{1}i_{2\;}\ldots i_{m-2}i_{m-1}} \end{array}$$

(4) $$\begin{array}{l} \overset{n_{j_{m-1}}}{\underset{i_{m}=1}{\sum }}p_{ii_{1}i_{2\;}\ldots i_{m-1}i_{m}}v_{ii_{1}i_{2\;}\ldots i_{m-2}i_{m-\;1}i_{m}} \end{array}$$

where *p* still denotes probabilities, *w* denotes weights, and *v* denotes outcome values from the modelled policy actions. Again, continuing from Section 3, traditional models call for unrealistic precision in the input information and thus, the quality of the modelling results deteriorates. Apart from the issues in Section 3, we here also have the additional complication of handling imprecision in probability estimates. Akin to imprecision in criteria weights and outcome values, it is desired to allow model makers to state their assessment of probabilities as intervals instead of fixed numbers, i.e., again not requiring unrealistic precisions in assessments. When also intervals are too hard to estimate, the possibility to use rankings of probabilities should be heeded as should the mixture of these options of expressibility, aiming at making the best possible use of the available probability information. The resulting models are computationally demanding and require advanced computer tools, in this paper the DecideIT decision-analytic software.

**Figure 3:**
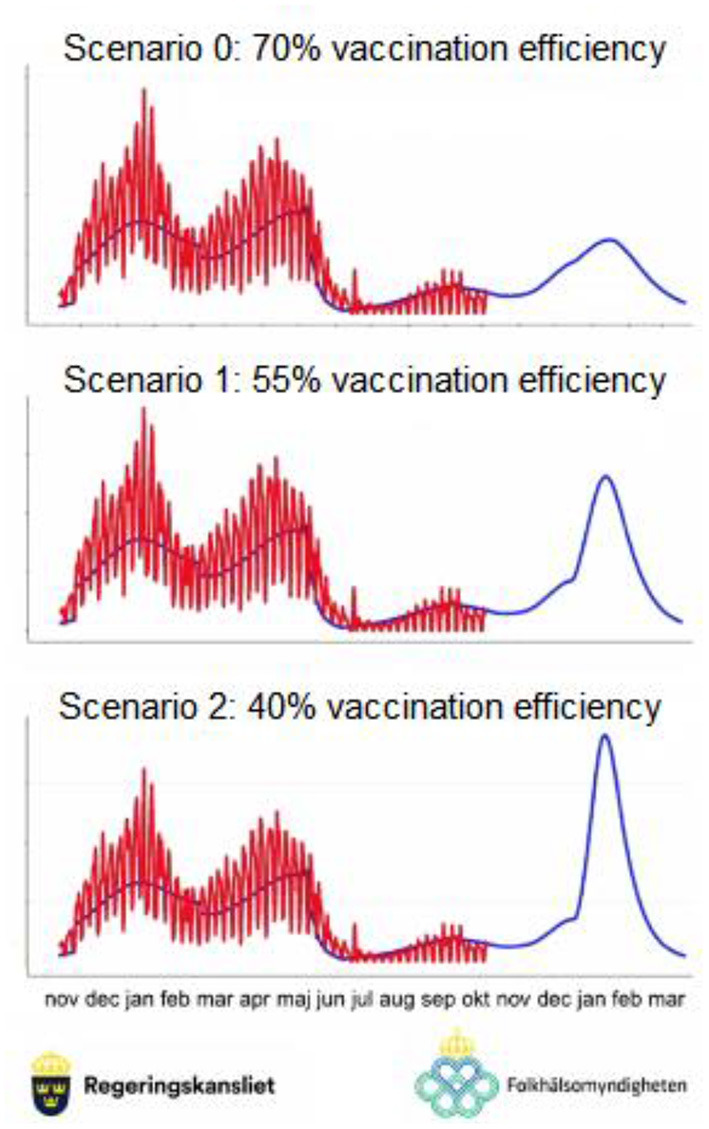
Swedish Government infection projections (joint press conference on Dec 21, 2021, together with the Swedish Public Health Agency).

**Figure 4:**
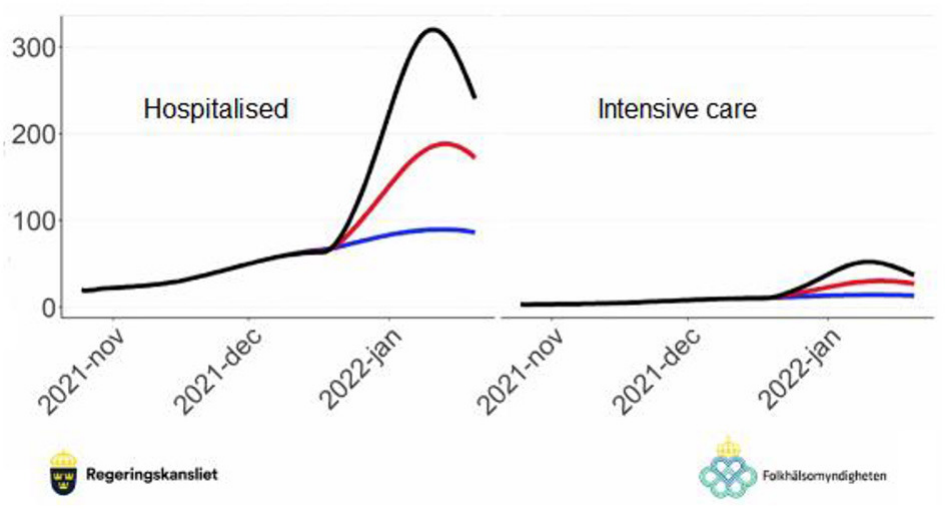
Prognosis on the number of new hospitalised cases per day **(left)** and the number of new cases in intensive care per day **(right)** in Sweden.

As an example, we look at the Swedish Government's analysis of the third wave of the Covid-19 pandemic, illustrated in Figures 3, 4. Sweden had adopted a fairly light lockdown policy, leading initially to larger casualties than neighbouring countries but also leading to much lower peaks later in the pandemic. At the end of 2021, it was feared that the, at the time, new SARS-CoV-2 strain Omicron, especially the BA.2 lineage, could break the population's vaccination barrier. The Government projected three possible scenarios for how efficient the, at the time, current vaccines offered to the population (AstraZeneca Vaxzevira, Pfizer/BioNTech Comirnaty, and Moderna Spixevax) would be when facing mutating SARS-CoV-viruses. The scenarios were named Scenario 0, 1, and 2, respectively and contained a vaccination efficiency of 70%, 55%, and 40% against mutated viruses. Figure 3 shows the prognosis by the Swedish Public Health Authority (*Folkhälso-myndig-heten*) where the x-axis contains months during 2021–2022 (up until March 2022) and the y-axis contains the number of people infected.

In Figure 3, the number of infected inhabitants in Sweden can be seen for each week from November 2020 to December 2021. The numbers are estimates since full population screening was not possible, and the heights of the bars show the width of the estimate for each week. The blue curve represents the average estimates up until December 2021 and is naturally the same in all scenarios. After December 2021, the blue line represents the projected number of infections in the future. Depending on the vaccination efficiency, the projected future curve has a very different shape and will put very different demands on the healthcare system. Thus, in order to protect the population different policy countermeasures are required depending on the probabilities of the scenarios.

Based on each of these three major scenarios, the increased pressure on the healthcare system as a whole was estimated (see Figure 4). On the left side, the number of estimated new hospitalised cases per day due to the mutated SARS-CoV-2 virus is shown for the three scenarios: Scenario 0 = 70% vaccine efficiency in blue, Scenario 1 = 55% in red, and Scenario 2 = 40% in black. Especially Scenario 2, with around 320 new hospitalised cases per day at its peak, would have put enormous pressure on the intensive care units of the major hospitals in Sweden, not least the respirators, if not major societal restrictions were imposed on the population.

**Figure 5:**
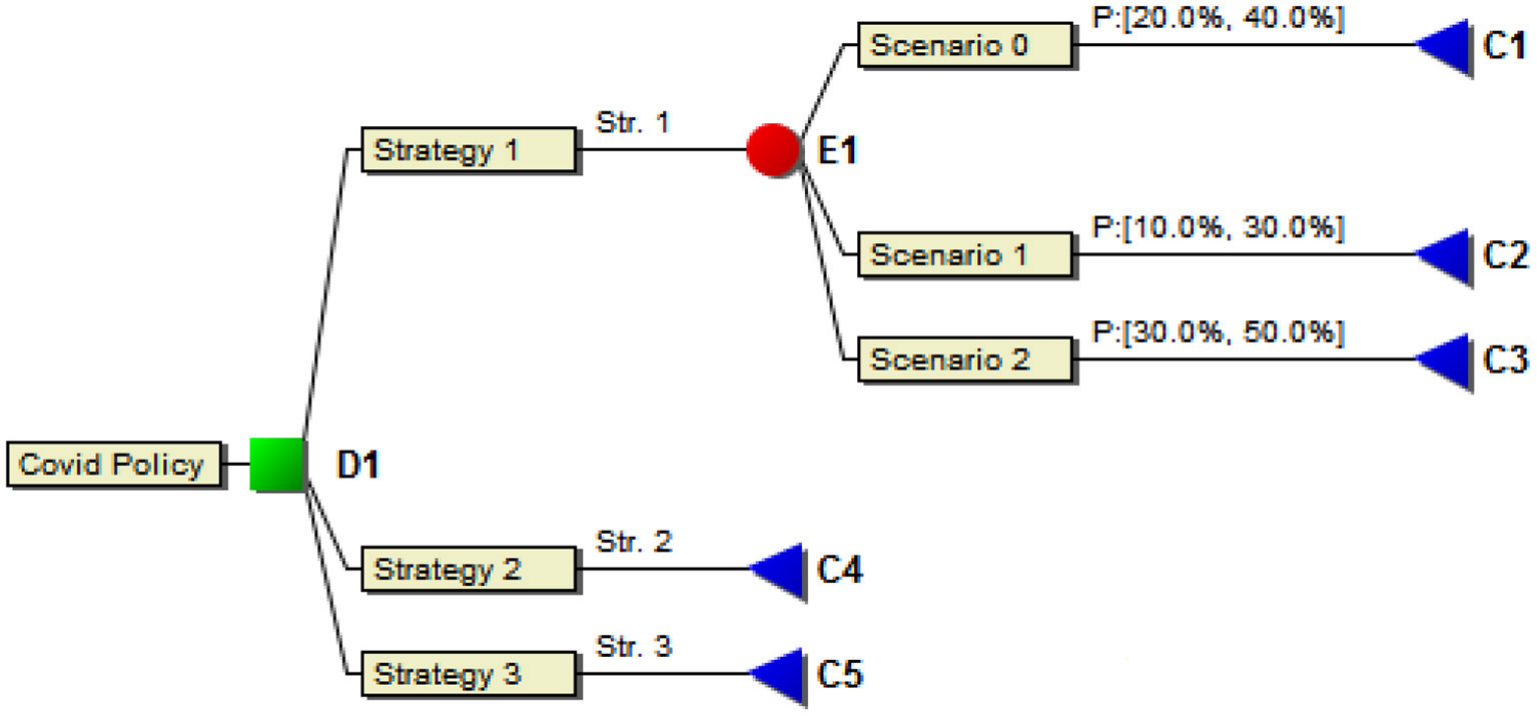
Strategy model with scenarios. For strategy 1, relying on the current vaccination programme, the three scenarios of Figures 3, 4, and their probability intervals, are shown.

This is a typical scenario analysis carried out for a particular criterion in the framework. Different criteria (or sub-criteria) can have different scenario analyses if required. Following the establishment of such scenarios, their respective probabilities should be estimated next. As mentioned above, this should not be done with unrealistic precision such as “*the probability of Scenario 2 occurring is 45%*” since the data is not known with any kind of precision like that. Instead, the framework we present allows the policy-makers to be as precise as they can but requires no more than that, instead allowing statements such as “*the probability of Scenario 2 occurring is between 30% and 50%*.” The latter statement is depicted in Figure 5 which shows estimates for the three scenarios as intervals representing the best judgements from a panel of experts with slightly differing views, all of which are accounted for in the policy decision model.

In the figure, Scenarios 0, 1, and 2 are handled by assigning probability intervals that embrace the assessments of a panel of experts given Strategy 1 which contained continuing vaccination of the entire population with at least three shots of vaccine per person. As is invariably the case in these kinds of modelling, the experts in the panel were not of the exact same opinion regarding the probabilities of each scenario occurring or the number of inhabitants admitted to intensive care. This was easily modelled by intervals that covered all the options of the expert panel. Each scenario is then subsequently assigned a cost in terms of stress for the healthcare system, cost of vaccination, etc. These are also preferably given as intervals since full numerical precision is unobtainable and thus unrealistic to require. The DecideIT decision analysis tool can be utilised for such analyses targeted at finding an optimal strategy given the societal circumstances, see Ekenberg et al. (25) for a demonstration of a non-probabilistic model evaluation. The additional complexity of the model in this paper is handled by allowing one scenario/event tree (such as in Figures 2, 5) connected to each criterion at the lowest (right-most) level (such as in Figure 1), although it is not necessary to have those for each criterion. For those that have, the scenario/event tree is evaluated first and an interval of expected values is obtained for each policy alternative. In the next step, the expected values of each (sub-)criterion are propagated in the criteria tree moderated by each criterion's weight interval. Finally, the weighted expected value of each policy option is obtained, and thereafter a set of sensitivity analyses commences. See Danielson et al. (19) for a discussion of the general features of the tool.

## 5. Discussion

Most countries were inadequately prepared when the Covid-19 pandemic hit the world in early 2020. First responses by governments were often some kind of lockdown, focusing on limiting the number of casualties. While it seemed reasonable at first, both for public health care and political reasons, it soon became evident that those lockdowns had severe societal effects when considering other aspects such as the economy, employment, education, and mental health. Slowly, models incorporating several societal criteria were developed. After some time, especially after experiencing and studying the SARS-CoV-2 virus' ability to mutate and its ease of travelling the world, it started to become evident that it was impossible to stop the spread worldwide, merely delay it (in order to, for example, flattening the peak demands on the healthcare system). As a recent case in point, witness the situation in China in January 2023 where prolonged lockdowns have not been able to contain the spread once they were lifted and where many countries, including Sweden, have reinstated PCR tests for incoming passengers travelling from China. Its mutability and all-year season make this virus all the more important to continuously monitor and model.

The next generation of policymaking decision analytic models must therefore contain considerably more detail in the sense of scenarios, for example regarding the efficiency of vaccination programmes or the impact on GDP of different levels of social distancing and lockdowns. These scenarios have probabilities to occur, and this tripartite policymaking model is the main contribution of this paper. It tries to cater for the needs of the next generation of modelling attempts that will, with a high likelihood, be required in the future. Since some of the authors are from Sweden, we have closely followed the strategies and analyses of the Swedish Government and the Swedish Public Health Agency during the pandemic. We have seen that their modelling attempts have become increasingly complex as time went by and more information became available. The same seems to be the case in many other European countries, while some such as Romania have returned to milder mitigation strategies regardless of vaccination efficiency due to socio-economic constraints. Further, the authors have scanned the literature but have not been able to find any pandemic (or other catastrophic) policymaking models able to cater for multiple criteria, multiple stakeholders, and probability assessments of scenarios in the same model. Such models should aid in having better societal preparedness when, not if, the next pandemic occurs—or the Covid-19 pandemic ups its pace once more.

## 6. Conclusions and further research

Our framework takes into consideration both epidemiologic estimations and socio-economic factors present in a complex, multi-faceted problem that needs to be managed. It also provides a starting point for designing future strategic communication in the public sphere which facilitates discussion towards informed policy, even in contexts of increased uncertainty. The main part of this is a multi-stakeholder and multi-criteria framework for eliciting attitudes, perceptions, and preferences amongst relevant stakeholder groups with regard to mitigation measures for catastrophic events, such as the worldwide Covid-19 pandemic crisis. The decision process is based on a recognition of the complex relationship between the different criteria involved and is supposed to support national strategies in dealing with pandemic emergencies and action plans, allowing for an alignment of overall objectives with perceptions and preferences of various stakeholder groups on priorities of actions, economic and political feasibility and others. It can thus be a basis for the development of policy recommendations together with policymakers, industry, and civil society at large. Since we assume that there is a heterogeneity of, and potential conflicts in, opinions of various stakeholder groups about disaster risk reduction measures, our methodology allows the development of recommendations for policies and strategies based on compromise solutions, thus increasing the quality, acceptability, legitimacy and implementability of the decision-making processes and their outcomes.

Using methods such as the suggested would, already in advance, provide insight into how to optimise hazard management options in relation to Covid-19-induced hazards. Further benefits include that stakeholders would become more aware of the availability of different options regarding each of the pertinent hazards to their communities, as well as the impact of their preferences on risk management and on the society at large, facilitating improvements in the resilience also regarding other future hazard events by providing a multi-stakeholder planning approach and contributing to more resilient regions. The policy options can be communicated with stakeholders and can also be used to gather feedback about how they recognise these. An important component here is also to combine it with a participatory approach of engaging different stakeholders, thereby increasing public understanding and reducing conflicts since various societal actors can acquire rich and deep insights into how their actions contribute to the escalation or mitigation of extreme hazards. A common understanding among different stakeholders is vital, in particular when restrictions are voluntarily undertaken.

Our research is an example of the application of systematic analysis of policy formation when the complexity of the Covid-19 risk mitigation and management policies is considered from the point of view of heterogeneity of opinions between different scientific disciplines such as epidemiology and economics and among various stakeholders. Thus our research could contribute and complement policy options that are based on the dominance of a few facts or considerations. The applications of this research methodology also facilitate a co-creation and compromise-building process for contested policy issues such as Covid-19 pandemic risk mitigation which involves stakeholders from various groups and identifying their preferences on various relevant factors for the health and socio-economic policy criteria.

## Data availability statement

The original contributions presented in the study are included in the article/supplementary material, further inquiries can be directed to the corresponding author.

## Author contributions

MD and LE provided the decision analytic expertise. NK handled most of the stakeholder interaction. AM provided the expertise on Romania and one of the three countries studied. MD, LE, NK, and AM contributed to the field work required to test the framework in real life. All authors contributed to the article and approved the submitted version.

## Footnotes

^1^ https://www.preference.nu/decideit/
